# Correlations between diffusion tensor imaging and levels of consciousness in patients with traumatic brain injury: a systematic review and meta-analysis

**DOI:** 10.1038/s41598-017-02950-3

**Published:** 2017-06-05

**Authors:** Jie Zhang, Rui-Li Wei, Guo-Ping Peng, Jia-Jia Zhou, Min Wu, Fang-Ping He, Gang Pan, Jian Gao, Ben-Yan Luo

**Affiliations:** 1 Department of Neurology & Brain Medical Centre, The First Affiliated Hospital, Zhejiang University, Hangzhou, China; 2 Department of Computer Science, Zhejiang University, Hangzhou, China; 3 Department of Rehabilitation, Hangzhou Hospital of Zhejiang CAPR, Hangzhou, China

## Abstract

Traumatic brain injury (TBI) often leads to impaired consciousness. Recent diffusion tensor imaging studies associated consciousness with imaging metrics including fractional anisotropy (FA) and apparent diffusion coefficient (ADC). We evaluated their correlations and determined the best index in candidate regions. Six databases were searched, including PubMed and Embase, and 16 studies with 701 participants were included. Data from region-of-interest and whole-brain analysis methods were meta-analysed separately. The FA-consciousness correlation was marginal in the whole-brain white matter (r = 0.63, 95% CI [0.47, 0.79], p = 0.000) and the corpus callosum (CC) (r = 0.60, 95% CI [0.48, 0.71], p = 0.000), and moderate in the internal capsule (r = 0.48, 95% CI [0.24, 0.72], p = 0.000). Correlations with ADC trended negative and lacked significance. Further subgroup analysis revealed that consciousness levels correlated strongly with FA in the CC body (r = 0.66, 95% CI [0.43, 0.89]), moderately in the splenium (r = 0.58, 95% CI [0.38, 0.78]), but insignificantly in the genu. In conclusion, FA correlates better with consciousness levels than ADC in TBI. The degree of correlation varies among brain regions. The CC (especially its splenium and body) is a reliable candidate region to quantitatively reflect consciousness levels.

## Introduction

Traumatic brain injury (TBI) is a common and severe type of injury, responsible for about one-third of injury deaths^1^. Country-level studies stated that its mortality rate ranged from 9 to 28.10 per 100,000 population per year^2^. Apart from risk of death, disorders of consciousness (DOC) caused by TBI also bring about enduring fears and despair. Regardless of injury severity, loss of or decreased consciousness is the common symptom of TBI. Some severe cases of TBI lead to persistent vegetative state (PVS) and minimally conscious state (MCS). The problem of impaired consciousness increases the burden of sufferers and their families and has baffled scientists and clinicians for decades.

Assessment of consciousness level is a critical step in clinical monitoring, risk assessment, and outcome prediction for patients with TBI. The Glasgow Coma Scale (GCS), first introduced in 1974 by Teasdale G *et al*., was created to simplify assessment of the depth and duration of impaired consciousness and coma^3^. It provides a structured and objective grading system for bedside assessment^4^. In clinical settings, such as the intensive care unit and neurosurgical unit, it has been used universally for more than 40 years. Other similar scales, including the Glasgow Coma Scale-Extended (GCSE)^5^, the Coma Recovery Scale-Revised (CRS-R)^6^, and the Disability Rating Scale (DRS)^7^, referred to the GCS and were derived from it. Due to its convenience and international approval, the GCS has become the most validated consciousness scale used in numerous research articles despite there being no gold standard for consciousness evaluation^8^.

However, the consciousness level detected by clinical scales is only the initial step. Hence, various advanced methods of diagnosis have been developed to explore additional information underlying the mystery of consciousness including functional magnetic resonance imaging, positron emission tomography, and event-related potential^9, 10^. Most studies using the above methods focused on the metabolism and activity of the cerebral cortex, and post-injury changes in subcortical structures were neglected. Recent research suggested that white matter fibres and circuits were also involved in the genesis and maintenance of consciousness^11–13^. Diffusion tensor imaging (DTI) is a well-suited tool to realise *in-vivo* visualisation of white matter^14^. Derived from diffusion-weighted imaging (DWI), DTI has several metrics to quantify both the degree and direction of water diffusion, including fractional anisotropy (FA), mean diffusivity (MD), axial diffusivity (AD), and radial diffusivity (RD)^15^. FA measures directional coherence of water diffusion within the tissue and reflects the degree of structural integrity and myelination of white matter^15, 16^. MD is referred as the rate of net diffusion of molecules, mathematically equivalent to averaged apparent diffusion coefficient (ADC)^14, 16^. ADC is often used to distinguish cytotoxic and vasogenic oedema^14, 15^. Similarly, AD and RD also describe the diffusion direction and magnitude^16^. With the help of the metrics above, neurologists are able to detect subtle subcortical pathological alterations of DOC^16^. In brief, DTI bridges this gap between impaired consciousness and white matter damage.

A previous systematic review highlighted the potential utility of DTI to be a biomarker in mild TBI patients by identifying the most vulnerable part of the corpus callosum (CC)^17^. The characteristics of DTI metrics in other subcortical regions such as the internal capsule (IC), centrum semiovale (CS), and thalamus have not been systematically summarised, and the subpopulation of moderate to severe TBI patients has not been investigated in a meta-analysis. Moreover, previous meta-analyses lacked correlations between DTI metrics and consciousness levels. Therefore, it is necessary to perform a new meta-analysis to evaluate the association of consciousness with DTI findings in TBI patients, which might contribute to accurate measurement of consciousness levels. Furthermore, subgroup analyses and meta-regression are planned to confirm whether multiple factors including subregions, demographic features, and study characteristics influence heterogeneity of specific outcomes.

## Results

### Study identification and selection

**Figure 1:**
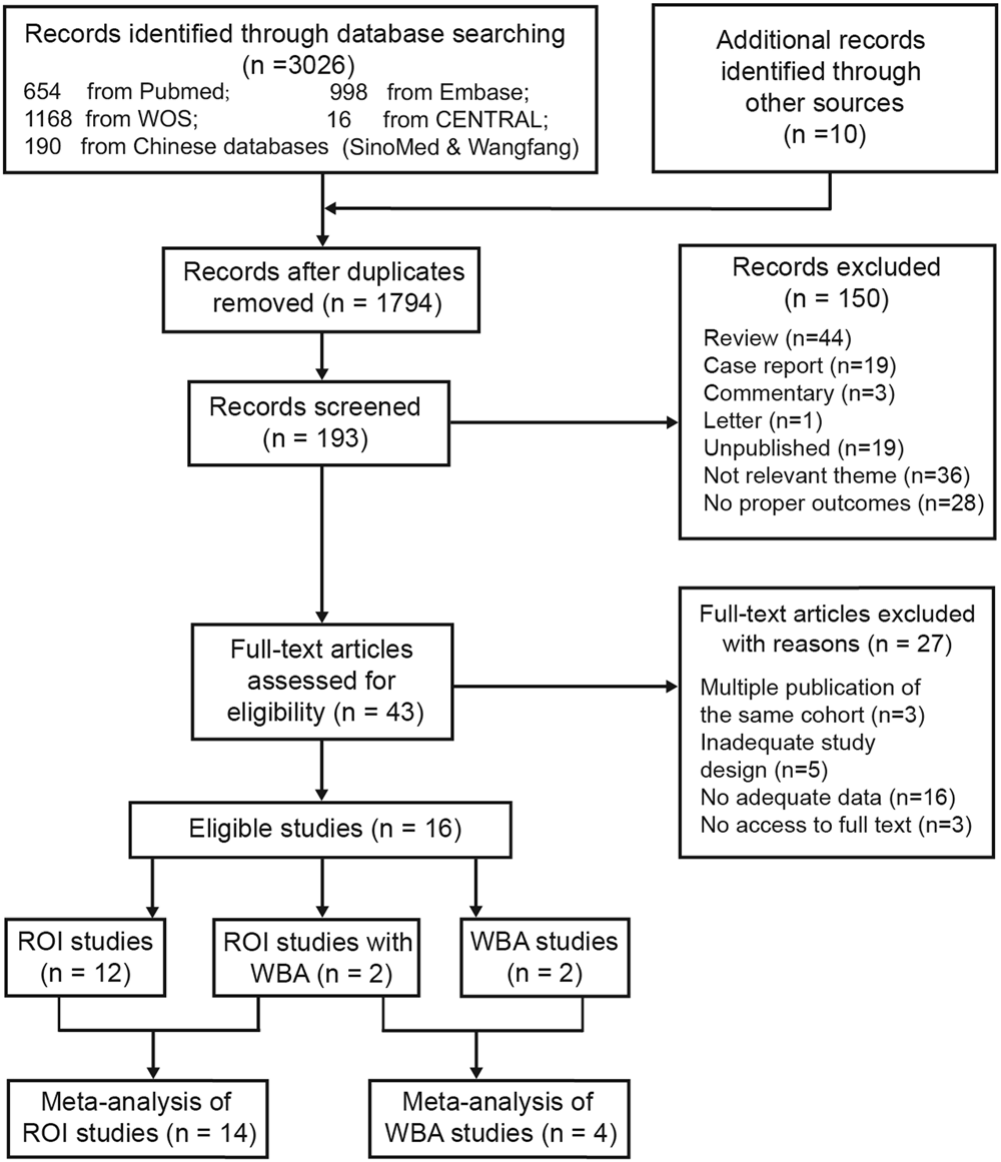
Flow diagram of the inclusion and exclusion of studies. ROI: region of interest; WBA: whole brain analysis.

The electronic database search identified 3026 records, of which 190 records were from Chinese databases (Fig. 1). In addition, 10 records were identified through other sources (1 from reference lists and 9 from Clinicaltrials.gov searches). After removing duplicates, 1794 titles were initially screened and 193 theme-related abstracts were selected for further screening. Next, 43 full-text articles were assessed for potential eligibility. Finally, 16 studies were included in this systematic review^18–33^. Fourteen studies reported region of interest (ROI) outcomes and 4 performed whole-brain analysis (WBA), 2 of which reported both the ROI and WBA. Noticeably, studies with different analytic methods were synthesised separately.

### Study characteristics and summarised MRI protocols

Characteristics of the 16 studies included are shown in Supplementary Table S1 ^18–33^. The included studies were published between 2004 and 2016. The number of participants per study ranged from 23 to 119, with a total number of 701. The mean age of patients in each study varied between 26.0 to 45.6 years, and only 2 studies included patients younger than 16 years old^20, 32^. Four studies reported years of education, ranging from 12 to 15 years^21, 27, 30, 31^. Most patients suffered TBI caused by motor vehicle accident, fall, assault, or other mechanisms. The median proportion of male patients was 72.70%. The severity of TBI included varied from mild to severe, and their corresponding levels of consciousness differed dramatically in the spectrum of TBI. While the mild TBI only involved transient concussion or slightly disturbed memory, PVS and MCS were common in severe TBI. Noticeably, patients with severe TBI were enrolled in each study included. Therefore, the result of this meta-analysis delivered more comprehensive information related to levels of consciousness. Few studies mentioned detailed injury positions. As to the imaging characteristics, ROI was the prevailing method for analysing imaging data, and the most studied area of white matter was the CC. FA was used to depict DTI findings in all of the studies, and most of them selected Spearman correlation coefficient to delineate the relationship between DTI indices and consciousness.

MRI protocols from all of the studies are summarised in Supplementary Table S2. More than half of the studies were performed on 3.0 Tesla scanners. Spin-echo echo-planar imaging (SE-EPI) was the routine sequence used for DTI.

### Risk of bias in the included studies

**Figure 2:**
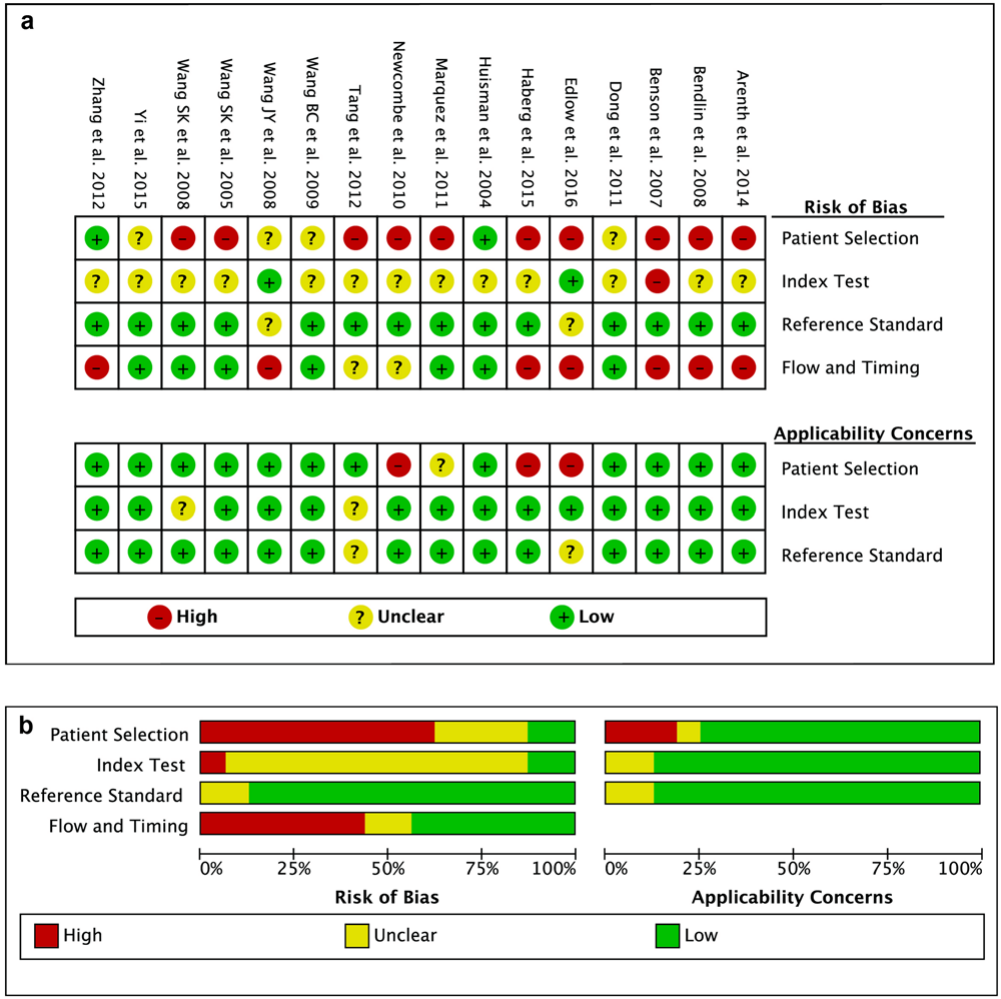
Assessment of risk of bias and clinical applicability. (**a**) Risk of bias summary. (**b**) Risk of bias graph.

The QUADAS-2 tool was applied to assess risk of bias and clinical applicability (see Fig. 2). Its original judgments are listed in the Supplementary Table S3. First, the tool suggested that the highest risk of bias was introduced during patient selection. Only 3 studies enrolled consecutive or random sample of patients^18, 29, 31^, and 5 only mentioned starting and ending times for enrollment^22, 24, 26, 32, 33^. Second, there was also a considerably high risk in the bias domain of flowing and timing, with 43.25% of studies presenting high risk. The interval between index tests and the reference standard was quite long in 5 studies^20–22, 30, 33^. Four studies did not analyse all of the included patients because of missing imaging data or dropouts^21, 22, 29, 31^. Overall, the included studies showed acceptable quality in most of the domains.

### Meta-analysis of correlations

After proper processing, all the 16 studies were accessible for data pooling. The r values for the study by Arenth *et al*.^30^ were calculated based on the r^2^ values from the original article. For the study by Newcombe *et al*.^25^, we omitted the points representing ischaemic/hypoxic injuries in the scatterplots and re-estimated the r values since we focused only on TBI. For special scales in which higher score presents poorer consciousness, opposite numbers of r values were calculated for final consistent presentation^28, 33^.

**Figure 3:**
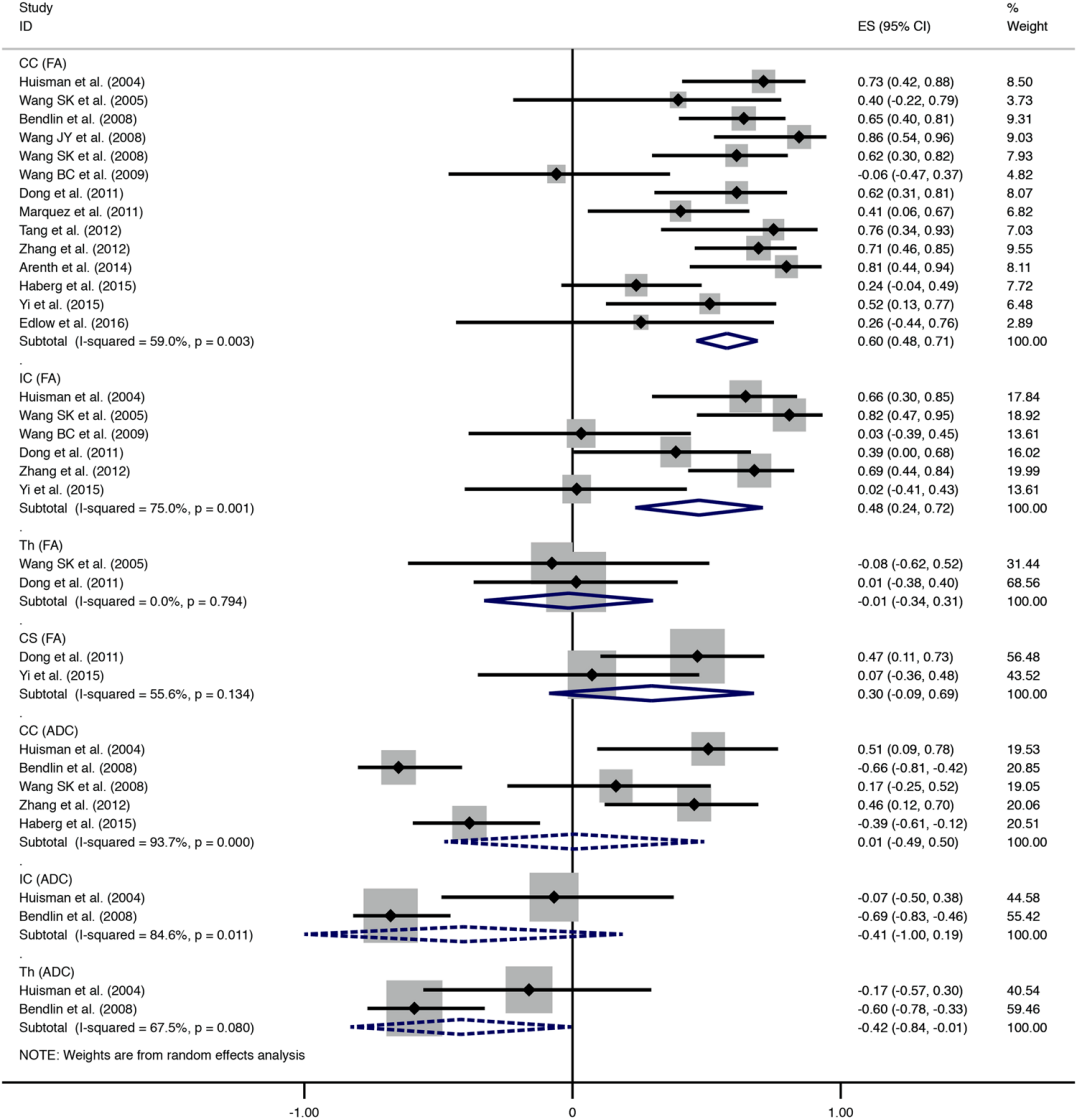
Summary forest plot comparing the FA/ADC-consciousness correlations of different brain regions from ROI studies. Solid-line diamonds represent overall r value of FA-consciousness correlations, and dotted diamonds are for ADC-consciousness correlations. FA, fractional anisotropy; ADC, apparent diffusion coefficient; CI, confidence interval; CC, corpus callosum; IC, internal capsule; Th, thalamus; CS, centrum semiovale.

Fourteen studies were included in the data synthesis of the ROI method, covering brain regions of the CC, IC, CS, and thalamus. The pooled correlations between DTI indices and consciousness were measured by r values after converting the Fisher’s z values back into correlation coefficients for presentation (Fig. 3). The pooled correlations with FA were marginally strong with excellent significance in the CC and moderate in the IC (CC: r = 0.60, 95% CI [0.48, 0.71], p = 0.000; IC: r = 0.48, 95% CI [0.24, 0.72], p = 0.000). However, in the CS and thalamus, there was no statistical significance for the correlations with FA (Thalamus: r = −0.01, 95% CI [−0.34, 0.31], p = 0.927; CS: r = 0.30, 95% CI [−0.09, 0.69], p = 0.130). In contrast, all the correlations with ADC in ROI studies were negative and lacked significance (Fig. 3).

**Figure 4:**
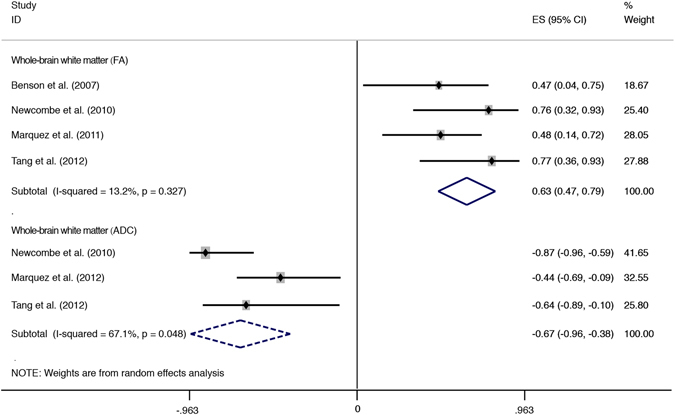
Forest plot showing correlations between consciousness and DTI indices of whole-brain white matter. Solid-line diamonds represent overall r value of FA-consciousness correlations, and dotted diamonds are for ADC-consciousness correlations. FA, fractional anisotropy; ADC, apparent diffusion coefficient; CI, confidence interval.

Four studies containing WBA results also were merged separately to investigate the correlations on the whole-brain level (Fig. 4). The 2 diffusion indices presented strong correlations with the consciousness level in the whole-brain white matter and achieved statistical significance (FA: r = 0.63, 95% CI [0.47, 0.79], p = 0.000; ADC: r = −0.67, 95% CI [−0.96, −0.38], p = 0.000).

As seen in Figs 3 and 4, the majority of pooled correlations presented moderate heterogeneity, e.g., correlations with FA in the CC (I^2^ = 59.0%, p = 0.003), IC (I^2^ = 75.0%, p = 0.001), CS (I^2^ = 55.6%, p = 0.134) and correlations with ADC in the whole-brain white matter (I^2^ = 67.1%, p = 0.048) or thalamus (I^2^ = 67.5%, p = 0.080). Mild heterogeneity was detected only in the correlations with FA in thalamus (I^2^ = 0.0%, p = 0.794) and the whole-brain white matter (I^2^ = 13.2%, p = 0.327). In addition, both the correlations with ADC in the CC and the IC were highly heterogeneous, I^2^ = 93.7% (p = 0.000) and I^2^ = 84.6% (p = 0.011), respectively. As a whole, correlations with ADC showed more pronounced heterogeneity than those with FA.

**Figure 5:**
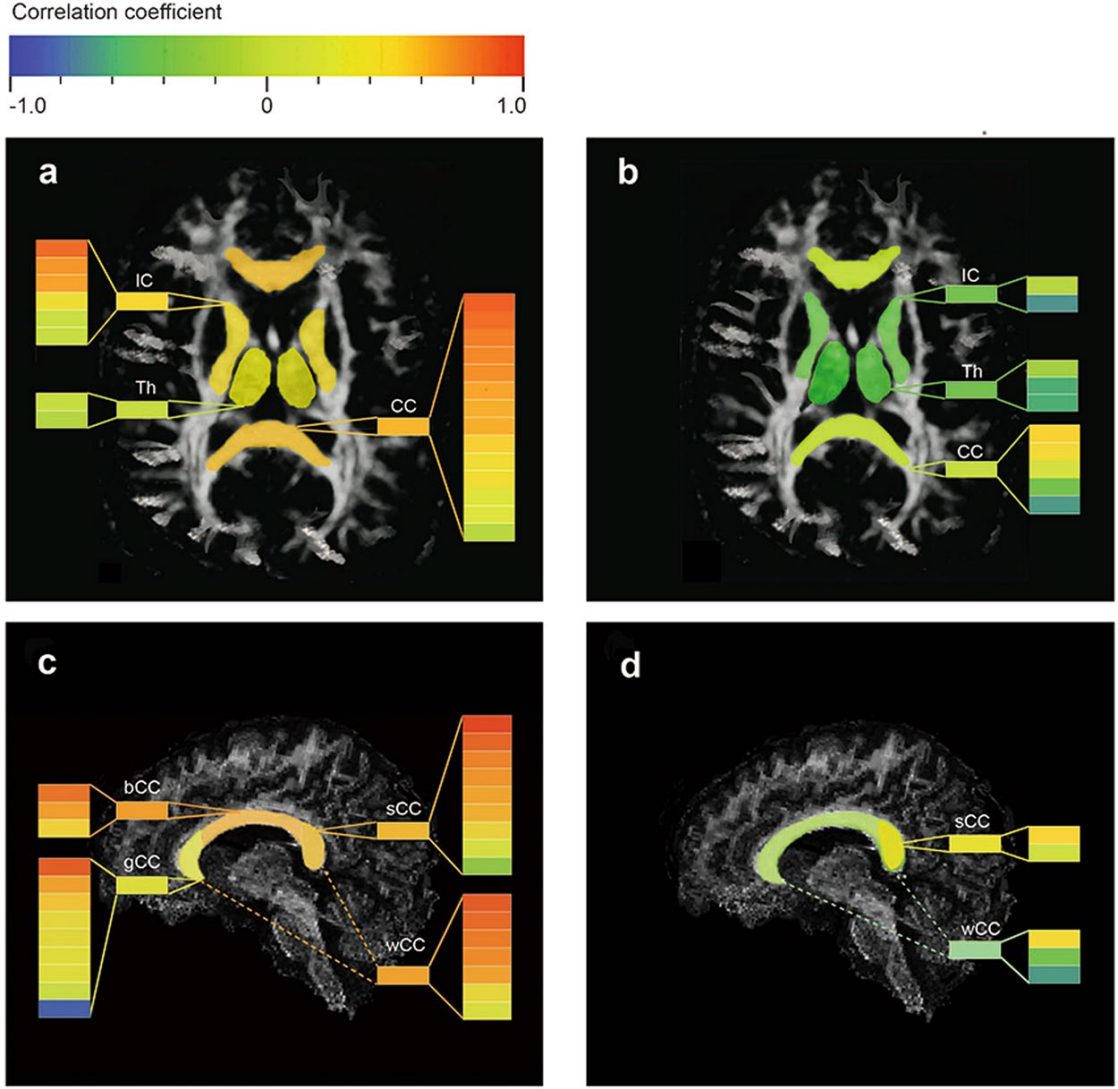
Colour-bar maps to visualize correlation coefficients in regions of interest. Positive correlations are shown in hot colours while negative correlations in cool colours. Each colour bar within a column represents a single study, and the colour bar before the column reflects pooled data after meta-analysis. (**a**) Colour-bar map reflecting FA-consciousness correlations in several brain regions. (**b**) Colour-bar map reflecting ADC-consciousness correlations in several brain regions. (**c**) Colour-bar map reflecting FA-consciousness correlations in subareas of the CC. (**d**) Colour-bar map reflecting ADC-consciousness correlations in subareas of the CC. CC, corpus callosum; IC, internal capsule; Th, thalamus; CS, centrum semiovale; sCC, splenium of corpus callosum; bCC, body of corpus callosum; gCC, genu of corpus callosum; wCC, whole corpus callosum.

Overall, the size and direction of correlation coefficients in respective ROIs are better visualised on the colour-bar maps (Fig. 5). Correlations with FA tended to be more positive, shown in hot colours. In contrast, cool colours were predominant in the colour-bar maps for ADC, presenting a trend of negative correlations with levels of consciousness.

### Sensitivity analysis, subgroup analysis, and meta-regression

**Figure 6:**
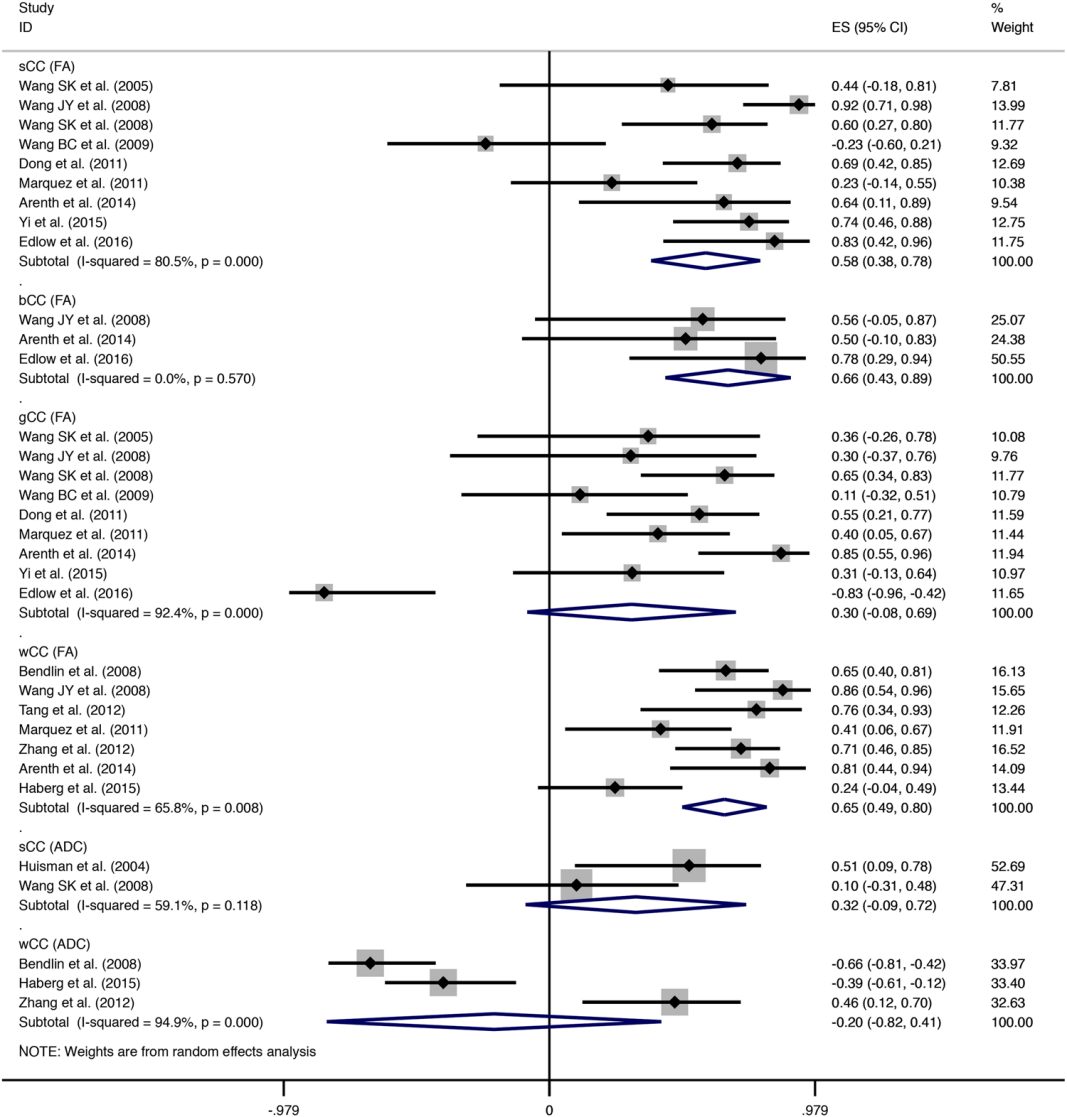
Subgroup analysis according to subareas within the CC from ROI studies. Solid-line diamonds represent overall r value of FA-consciousness correlations, and dotted diamonds are for ADC-consciousness correlations. FA, fractional anisotropy; ADC, apparent diffusion coefficient; CI, confidence interval; sCC, splenium of corpus callosum; bCC, body of corpus callosum; gCC, genu of corpus callosum; wCC, whole corpus callosum.

Since most of the correlations above showed obvious heterogeneity, it was necessary to seek the sources. Out of the total 16 studies, 14 reported the outcome of correlations with DTI indices in the CC, including sufficient number of studies for further analysis^18, 19, 21–24, 26–33^. In contrast, other ROIs, such as the CS and thalamus, did not have enough published data from their subareas. First, we performed subgroup analysis according to subareas of the CC. The CC could be divided further into several segments including genu, body, and splenium according to Witelson’s classification^34^. Nine studies investigated correlations with FA in subareas of the CC^19, 22–24, 26, 27, 30, 32, 33^, and 2 studies reported the ADC-consciousness correlation in those areas^18, 23^. For correlations with FA, obvious decline of heterogeneity was detected only in the CC body (I^2^ = 0.0%, p = 0.570; Fig. 6), showing almost no heterogeneity. For correlations with ADC, moderate heterogeneity was found in the splenium (I^2^ = 59.1%, p = 0.118), notably reduced from its original level of high heterogeneity (I^2^ = 93.7%, p = 0.000). Moreover, in the majority of subareas within the CC, pooled correlations with FA retained robust significance even after subgroup analysis (splenium: p = 0.000; body: p = 0.000). The only correlation without significance was in the genu (p = 0.122). Furthermore, we found the strength of correlations varied within the subareas: strong in the body (r = 0.66, 95% CI [0.43, 0.89]), moderate in the splenium (r = 0.58, 95% CI [0.38, 0.78]), and weak in the genu (r = 0.30, 95% CI [−0.08, 0.69]).

Sensitivity analysis was also attempted by excluding Edlow’s study, considering its control group was not well designed. For the correlation with FA in the genu, significant decline of heterogeneity was detected from a high level (I^2^ = 92.4%, p = 0.000) to a moderate level (I^2^ = 58.4%, p = 0.019), and the pooled correlation in the genu achieved pronounced significance (r = 0.49, 95% CI [0.31, 0.68], p = 0.000).

Furthermore, we performed meta-regression to evaluate more potential factors (Table 1). The factors can be divided into demographic features (proportion of male sex, mean age, and nationality of the TBI group) and study characteristics (delay to DTI scan and length of interval between DTI and GCS). Both univariate and multivariate meta-regression indicated that none of the above factors contributed to the heterogeneity of meta-analysis (all p values > 0.05). Lastly, we were disappointed that we were unable to find relevant individual data to assess the potential heterogeneity from average injury severity and specific injury mechanism, and thus could not perform the analysis.

**Table 1:** Summary of meta-regression analyses for the potential source of heterogeneity.

Heterogeneity factors		Coefficient	SE	95% CI	P
**Univariate meta-regression**					
Proportion of male		0.034	1.505	(−3.320, 3.389)	0.982
Mean age		−0.005	0.017	(−0.042, 0.033)	0.786
Interval between DTI and scales	Long	0.193	0.210	(−0.269, 0.656)	0.378
Delay to DTI scan	Acute	−0.134	0.368	(−0.953, 0.686)	0.724
	Chronic	−0.263	0.404	(−1.163, 0.636)	0.528
Nation	China	−0.351	0.373	(−1.183, 0.480)	0.369
	Norway	−0.671	0.450	(−1.675, 0.333)	0.167
	US	−0.137	0.379	(−0.982, 0.706)	0.723
**Multivariate meta-regression**					
Proportion of male		−0.314	2.980	(−7.970, 7.341)	0.920
Mean age		−0.001	0.053	(−0.143, 0.130)	0.905
Delay to DTI scan	Acute	0.063	0.566	(−1.393, 1.519)	0.915
	Subacute	0.050	0.589	(−1.464, 1.563)	0.936
Nation	China	−0.322	0.862	(−2.538, 1.894)	0.724
	Norway	−0.617	0.874	(−2.865, 1.630)	0.511
	US	−0.128	0.645	(−1.787, 1.531)	0.851

SE, standard error; CI, confidence interval.

### Analysis for publication bias

**Figure 7:**
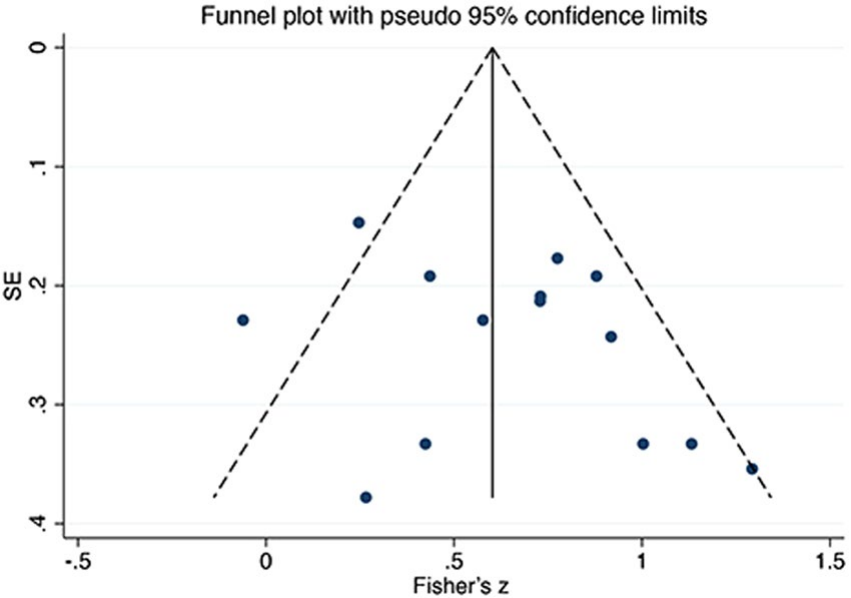
Funnel plot of pooled correlations between FA and consciousness in the CC. To draw the funnel plot, correlation coefficient values were converted into Fisher’s z values which fitted normal distribution. SE, standard error.

We detected the publication bias for correlations between FA and levels of consciousness in the CC, since it was the only outcome including more than 10 studies. The funnel plot showed no obvious asymmetry (Fig. 7) and Egger’s test did not demonstrate any significant bias (p = 0.208, p > 0.1). Therefore, no obvious publication bias was detected for this primary outcome.

## Discussion

The present systematic review and meta-analysis investigated correlations between DTI indices and consciousness and drew comparisons between different brain regions and indices. We found that FA tended to correlate positively with levels of consciousness, while ADC had a trend toward negative correlations. Additionally, FA presented more significant correlations and less pronounced heterogeneity than did ADC. Moreover, the degree of correlations varied between different regions. The correlation with FA was marginally strong in the CC, moderate in the IC, and mild in the CS and thalamus. Strong correlations were observed in the whole white matter, whether the DTI index was FA or ADC. Inversely, both the correlations with FA and ADC were mild in the thalamus. We were disappointed not to be able to analyse pooled correlations with the number of fibres or the volume of white matter because of lack of sufficient number of studies including those data.

To make the results generalizable, we included studies with various post-injury intervals, severity, and other factors in this systematic review and meta-analysis. However, obvious heterogeneity was detected in the majority of pooled effect estimates. To mitigate this effect, multiple methods were performed to seek responsible sources of heterogeneity. This strategy proved to be effective for reducing heterogeneity after subgroup analysis of subareas within the CC and removing poorly designed studies. However, factors including delay to scan, interval between DTI and scales, and demographics did not contribute to statistical heterogeneity.

Previous DTI-related meta-analyses used a visualised method named activation likelihood estimate (ALE) to locate regions with white matter abnormalities^35, 36^. Another meta-analysis pooled FA values to investigate the most vulnerable region^17^. Briefly, all of them focused on seeking out the abnormal regions. Compared to previous systematic reviews or meta-analyses, our study has several advantages. First, we related abnormal imaging metrics with clinical measures of consciousness. To our knowledge, this is the first meta-analysis to investigate correlations between imaging findings and clinical outcomes. Second, we compared the differences between multiple indices including FA and ADC, and also compared ROIs other than the CC. FA value is a highly sensitive but fairly nonspecific biomarker^37^, and hence, the combination of the ADC result could be more convincing. We also investigated the characteristics of different brain regions by subgroup analysis to determine the best correlations. Lastly but critically, the research subjects of our study are significantly distinct from the previous publication which included only mild TBI.

Since the clinical manifestation and pathology of white matter damage are dramatically different between mild and severe TBI, these data might have different clinical value. A critical factor for determining the consciousness of the individual mental state is how well the brain activity corresponding to one particular state can be distinguished or separated from alternative possibilities^38^. Clinical scales provide simple but typical tasks, such as verbal or motor responses, to provoke the distinguished brain activity. The GCS assesses the level of consciousness with separate ratings of the 3 components^39^, and our results showing correlations with the clinical scales delivered more information about the association between white matter structure and consciousness.

In light of pronounced sensitivity of DTI in detecting damaged tissue, it is feasible to analyse a full spectrum of severity in TBI patients^40^. As mentioned above, FA measures the structural integrity of white matter fibres. The higher the FA value, the more intact the fibres. Moreover, the level of consciousness is strongly correlated with FA in the CC from the aspect of our study, consistent with theories provided by previous studies. For instance, Rutgers *et al*. also found that patients with severe TBI had significantly lower FA in the CC compared with mild TBI patients and control subjects^41^. We proposed several hypotheses to interpret the importance of the CC. First, traumatic axonal injury (TAI) caused by acceleration or deceleration forces is a frequent mechanism of impaired consciousness in patients with TBI^41^. The CC has the largest commissural white matter bundle in the brain which makes it exactly one of the most vulnerable regions of TAI^42^. It was reported that consciousness was disrupted in patients whose corpus callosa were resected^43^. Two independent streams of conscious awareness appeared in each hemisphere that could not be unified normally. This finding is supported by current evidence that complete section of the corpus callosum has been reported to result in loss of inter-hemispheric resting-state functional connectivity^44^. Second, the damaged CC may affect the connectivity of the default mode network (DMN). Recent research suggested a critical role for the DMN in the genesis of awareness^45^. FA can be used to measure the structural integrity, and the abnormal DMN is more closely related to impaired consciousness compared with the global white matter skeleton^12^. It is said that MRI changes in CC might be related to reduced functional connectivity of the posterior cingulate cortex/precuneus in the DMN, leading to consciousness disturbance in encephalopathy^46^. Third, traditional imaging studies have indicated that the CC is unevenly affected in TBI, suggesting that TAI is more commonly shown in the splenium^42, 47^. Moreover, FA values of splenial fibres also correlated with blood oxygenation level dependent (BOLD) activation and inter-hemispheric synchronisation^48, 49^. Accordingly, these studies support the outcome of our subgroup analysis that the splenium and body showed much stronger correlations with FA than the genu.

As for the whole-brain results, our meta-analysis found a strong association of whole-brain FA with clinical consciousness scales. This finding is supported by a longitudinal study that revealed a significant positive correlation between the improving consciousness disorder and the number of voxels with an FA value >0.5 in the whole brain^50^. This phenomenon could be interpreted to indicate that the average integrity of global white matter may be involved in the maintenance of consciousness; hence, the WBA outcome seemed to have a better correlation.

From another aspect, the degree of post-traumatic brain oedema is one of the main determinants of neurological outcome and survival^51^. The disruption of the blood-brain barrier (BBB) and formation of vasogenic oedema reflect secondary injury after TBI^52^. In our research, the strong negative correlation between clinical outcomes and ADC in the whole white matter indicates that global vasogenic oedema after injury might contribute to impaired consciousness. The relationship between the GCS and whole-brain ADC values is supported by DWI studies detecting non-visible TBI lesions^53, 54^.

Moreover, we are also interested in the potential time-varying relationship between DTI metrics and clinical outcomes. A longitudinal post-traumatic study revealed that mean FA values decreased significantly at the second DTI scan^50^. In the chronic phase, multiple mechanisms may underlie this finding, including demyelination, axonal disconnection, astrogliois, or damage to intracellular cytoskeleton and neurofilaments^55^. However, meta-regression analyses in our study did not detect any heterogeneity from various lengths of delay to DTI scan, which is probably because there were not enough studies with long-delay scans to present their valid discrepancy. Moreover, other undetected heterogeneous factors such as injury severity and mechanisms might confound the underlying phenomenon.

Admittedly, there were some obstacles and limitations of the present study. First, several studies provided correlation coefficients without elucidating their definite types^18, 21, 28, 31^. A transformation process with a special formula should be performed if these were Pearson correlation coefficients. For the purposes of this study, we regarded Spearman correlation as the default type for the unknown data, which might yield a slight loss of efficiency and introduce the bias of estimation if bivariate normality assumptions are met. However, the score of clinical scales such as the GCS is an ordinal variable and scale measurements often feature a non-normal distribution^56^. Therefore, Spearman correlation is more suitable for this data type. Second, studies that did not analyse DTI-consciousness correlations had to be excluded, and few studies included mentioned the insignificant correlations in specific ROIs without reporting their values. It is better to request the unpublished data of correlation coefficients; however, it was difficult to derive them all. Nevertheless, no obvious publication bias was detected by statistical methods. Third, although all of the included studies designed control groups, few studies reported r values of control groups because most of them were not significant. The conclusion would be more convincing if pooled r values of control groups were available. Lastly, the clinical scales for rating consciousness were not consistent, which could increase the heterogeneity. Future studies should include more participants with less selection bias and provide a cut-off FA value of ROIs. We hope DTI could shed light on the precise diagnosis of consciousness impairments.

In conclusion, this systematic review and meta-analysis demonstrates that DTI indices possess the potential to reflect levels of consciousness in TBI patients, and FA correlates better with clinical scores than ADC. Comparing whole-brain and regional analyses, the impaired whole-brain white matter has the most stable association with levels of consciousness. In addition, within the CC, the splenium and the body showed much stronger FA-consciousness correlations than the genu; hence, they are regarded as the potential regions to quantify the degree of consciousness disorders. In addition, delay to scan, interval between DTI and clinical evaluation of scales, and demographic factors contribute no statistical heterogeneity, making the interpretation of correlations more convincing.

## Methods

This systematic review and meta-analysis was performed in accordance with the Preferred Reporting Items for Systematic Reviews and Meta-Analyses (PRISMA) Statement^57^.

### Search strategy and inclusion criteria

Electronic searches of studies published between January 1, 1994 and January 8, 2017 were performed in PubMed, Embase, the Cochrane Central Register of Controlled Trials (CENTRAL), Web of Science and two Chinese databases (SinoMed and Wanfang Data). We did keyword and MeSH searches for our theme. We used combined Medical Subject Heading (MESH) terms, key words and their synonyms related to “diffusion tensor imaging”, “traumatic brain injury” and “consciousness disorders”. The complete strategies are listed in the supplementary material. We also manually checked the reference lists of retrieved articles and searched Clinicaltrials.gov for studies from other sources.

Studies were considered for inclusion if they: (1) were done in patients with TBI; (2) investigated the relationship between DTI and consciousness; (3) were published as full papers in peer-reviewed scientific journals. Exclusion criteria were: (1) studies that only included pediatric patients; (2) the number of included patients was less than five; (3) further publications from the same cohort.

### Data extraction and quality assessment

Two reviewers (J.Z. and J.J.Z.) independently screened records based on titles and abstracts according to the inclusion/exclusion criteria. Then, full text articles were assessed for eligibility by 2 reviewers (J.Z. and R.L.W.). Disagreements regarding selection were resolved by discussion among all authors. Data were extracted into several tables by 1 reviewer (M.W.), and two reviewers (R.L.W., J.J.Z.) checked the extracted data for agreement.

The risk of bias in the included trials was assessed independently by 2 reviewers (R.L.W. and B.Y.L.), according to the Quality Assessment of Diagnostic Studies 2 (QUADAS-2) instrument provided by the Cochrane Handbook for Diagnostic Test Accuracy Reviews^58, 59^. Discussions or meetings were arranged to minimise differences when disagreement between 2 reviewers arose. The figures for risk of bias were made using RevMan 5.3 (Review Manager 5.3, Cochrane Informatics and Knowledge Management Department).

### Outcomes

All of the studies measured levels of consciousness using clinical scales on admission and at discharge. The scales related to consciousness include the GCS, the GCSE, the CRS-R, and the DRS. The GCS is characterised by 3 components, i.e., motor, verbal, and eye responses. The GCSE is an extension of the traditional GCS^5^, whose supplementary scoring system is helpful in the acute identification of mild TBI^60^. The DRS is also used to evaluate the impairment of consciousness, of which the first 3 items are a slight modification of the GCS and a score above 22 indicates PVS^7^. The CRS-R is composed of 6 subscales, and is the most acceptable rating in patients with DOC^6, 61^. Noticeably, in above scales except the DRS, a higher score presents better consciousness.

The primary outcome of our study is the correlation coefficient between FA and levels of consciousness measured by scales. For secondary outcomes, we investigated the correlation between ADC and levels of consciousness. Correlations with AD and RD were inappropriate to pool together because they were not reported by enough studies.

### Statistical analysis

All the data in this quantitative analysis were the result of various correlations. Spearman correlation was the most common method. Given that outcomes were measured by clinical scoring, it is appropriate to use Spearman rank correlation. Data reported by Pearson correlation would be converted into Spearman correlation coefficients by a specific formula: ${r}_{Spearman}=\frac{6}{\pi }{\sin }^{-1}\frac{{r}_{Pearson}}{2}$ ^62^. If the correlation coefficient was reported without a definite type, Spearman correlation was regarded as its default type. Next, Fisher transformation was used to convert each correlation coefficient into an approximately normal distribution. Thus, the standard error (SE) of Fisher’s z values and 95% CIs could be calculated to evaluate the degree of variation^63, 64^. Finally, the Fisher’s z values would then be converted back to correlations for presentation^63, 64^.

Meta-analyses were conducted using StataSE 13 (Stata-Corp, College Station, TX, USA). The heterogeneity across each effect size was evaluated with Q-statistics and the I^2^ index. We considered I² > 50% to be pronounced heterogeneity. The method of inverse variance using random-effect models was chosen for all the meta-analyses. The pooled correlation coefficients and 95% CIs were calculated to test the results of different trials. A P value < 0.05 was considered as clinical significance. Subgroups were divided according to brain regions and DTI indices. We also performed meta-regression to analyse more potential heterogeneous factors. The risk of publication bias was detected for the outcome with more than 10 studies. We used StataSE 13 to make funnel plots and performed Egger’s test to quantify the publication bias. Significant publication bias was defined as p value < 0.1 being statistically significant.

This study has been submitted to PROSPERO and its registration number is CRD42016038547.

### Data availability statement

The datasets generated during or analysed during the current study are available from the corresponding author on reasonable request.

## Electronic supplementary material

Online Supplement
