# Supplementary material for: Correlations between diffusion tensor imaging and levels of consciousness in patients with traumatic brain injury: a systematic review and meta-analysis
Source: Sci Rep. 2017 Jun 5;7:2793. doi: 10.1038/s41598-017-02950-3 (PMC5459858; doi:10.1038/s41598-017-02950-3)
Supplement: Supplementary file 1 — Online Supplement [file 41598_2017_2950_MOESM1_ESM.pdf]

Correlations between diffusion tensor imaging and levels of consciousness in patients with traumatic brain injury: a systematic review and meta-analysis

Jie Zhang[1], Rui-Li Wei[1], Guo-Ping Peng[1], Jia-Jia Zhou[1], Min Wu[1], Fang-Ping He[1], Gang Pan[2], Jian Gao[3], and Ben-Yan Luo[1]

1 Department of Neurology & Brain Medical Centre, The First Affiliated Hospital, Zhejiang University, Hangzhou, China
2 Department of Computer Science, Zhejiang University, Hangzhou, China.
3 Department of Rehabilitation, Hangzhou Hospital of Zhejiang CAPR, Hangzhou, China.

Supplementary-Search Strategy

1.Pubmed

1# DTI[Title/Abstract] OR Diffusion Tensor Imaging[Title/Abstract] OR tensor[Title/Abstract] OR TBSS[Title/Abstract] OR tract-based spatial statistics[Title/Abstract] OR tractography[Title/Abstract] OR Diffusion Tensor[Title/Abstract] OR fractional anisotropy[Title/Abstract] OR "Anisotropy"[Mesh] OR "Diffusion Tensor Imaging"[Mesh] OR diffusion tensor MRI[Title/Abstract]
2# traumatic brain injury[Title/Abstract] OR traumatic brain injuries[Title/Abstract] OR TBI[Title/Abstract] OR "Brain Injuries, Traumatic"[Mesh]

3# 1# AND 2#

2.Embase

#1 DTI:ab,ti OR 'diffusion tensor imaging':ab,ti OR tensor:ab,ti OR tbss:ab,ti OR 'tract-based spatial statistics':ab,ti OR tractography:ab,ti OR 'diffusion tensor':ab,ti OR 'fractional anisotropy':ab,ti OR 'diffusion tensor mri':ab,ti OR ’diffusion tensor imaging'/exp OR 'tractography'/exp OR 'fractional anisotropy'/exp

#2 'traumatic brain injury'/exp OR 'traumatic brain injury':ab,ti OR 'traumatic brain injuries'/exp OR 'traumatic brain injuries':ab,ti OR TBI:ab,ti

#3 'human'/de
#4 #1 AND #2 AND #3

3.CENTRAL (Cochrane Library)

#1 MeSH descriptor: [Diffusion Tensor Imaging] explode all trees
#2 MeSH descriptor: [Anisotropy] explode all trees
#3 DTI or Diffusion Tensor Imaging or tensor or TBSS or tract-based spatial statistics or tractography or Diffusion Tensor or fractional anisotropy or diffusion tensor MRI:ti,ab,kw (Word variations have been searched)

#4 traumatic brain injury:ti,ab,kw (Word variations have been searched)
#5 MeSH descriptor: [Brain Injuries] explode all trees

#6 #1 OR #2 OR #3
#7 #4 OR #5
#8 #6 AND #7

4.Web of Science

1# TS=(DTI OR “Diffusion Tensor Imaging” OR tensor OR TBSS OR “tract-based spatial statistics” OR tractography OR “fractional anisotropy” OR “diffusion tensor MRI”)
2# TS= (“traumatic brain Injuries” OR “traumatic brain Injury” OR TBI)

3# 1# AND 2#

Supplementary Table S1. Characteristics of the included studies

Study	Nation	Number of participants (Exp/Ctr)	Age (years) (Exp/Ctr)	Years of education	Proportion of male in TBI group (%)	Mechanisms/Cause	Severity	Postinjury intervals for assessment (Scanning/Scale) （Time since injury）	Scale	Assessed areas	DTI indices	Correlation type
**ROI studies**												
Huisman et al., 2004	Switzerland	20/15	30±10/35±10	-	75.00	DAI	Mild to severe: GCS 8.7±3.7	Scan: ≤7 days; Scale: mean 18 days	GCS	CC, IC, putamen	FA, ADC	-
Wang SK et al., 2005	China	12/13	40 (20-64) / 38 (25-58)	-	58.33	Traffic accident / contusion injury: 10; fall injury: 2	Mild to severe: GCS 6-14	Scan and scale: ＜14 days	GCS	CC, IC	FA	Spearman
Wang JY et al., 2008	US	11/12	26±8.1 (16-37)	-	-	DAI	Severe TBI: 4.4±2.1 (3-8); GOSE: 4.4±2.2 (1-8)	Scan: 6.7±4.2 (0-15) days; Scale: 8.2±1.6 (6-11) months	GCSE	CC, fornix, PVF, PDF, Ppar, Pocc	FA	Spearman
Wang SK et al., 2008	China	25/20	37 (20-60) / 36 (21-60)	-	64.00	DAI	Mild to severe	Scan and scale: 1-15 days	GCS	CC	FA, ADC	Spearman
Wang BC et al., 2009	China	22/14	37 (20-58) / 32 (21-53)	-	72.73	18 MCA/Contusion injury, 4 Fall	Mild to severe: mild 14, moderate 2, severe 6	Scan: ＜7 days	GCS	CC, IC	FA	Pearson
Dong et al., 2011	China	26/20	41.6 (19-60) / 39.8 (20-59)	-	73.08	18 MCA, 4 Fall, 4 Contusion injury	Mild to severe: GCS 5-13	Scan: ＜20 days	GCS	CC, IC, thalamus, centrum semiovale	FA	Spearman
Zhang et al., 2012	China	59/60	37.88±14.39 / 36.88±16.14	-	57.63	Traffic accident: 27, contusion injury: 13, fall injury: 19	Mild to severe	Scan: Acute＜7 days, Subacute 7-30 days, 3rd follow-up 1-3 months	GCS	CC, IC, midbrain	FA, ADC	Spearman
Arenth et al., 2014	US	12/12	32.3±12.0(18-54)/29.8±10.8(19-50)	14.7±2.3(10-18)/16.2±3.1(12-22)	83.33	8 MVA, 2 Fall, 2 Other injury mechanisms (1 Sports injury, 1 Falling object)	Mild to severe: 5 mild, 2 moderate, 5 severe; the highest GCS score in the 1st 24 h: 10.5±4.2	Scan: 1.7±0.58(1-3) years; Scale: in the 1st 24 hours	GCS	CC	FA	Spearman
Haberg et al. , 2015	Norway	49/50	29.2±12.1/32.7±12.1	11.9±2.3/12.16±2.16	73.47	23 MVA, 22 Fall, 4 Other injury mechanism	Moderate to severe: GCS: 8.8±3.6;	Scale (GCS): at time of arrival in the emergency	GCS & GCSE	CC	FA, ADC, WM volume	-

							GCSE: 6.7±1.4	room; Scans and GCSE: acquired in the chronic phase (2.8±1.1 years)				
	Yi et al., 2015	China	22/12	45.6 (11-71) /42.3 (25-56)	-	72.73	Traffic accident: 17, contusion injury: 2, fall injury: 3	Mild to severe: mild 7, moderate 5, severe 10	Scan: <10 days	GCS	CC, IC, centrum semiovale	FA	Spearman
	Edlow et al., 2016	US	10/1	36.2±19.7 (16-77)	-	72.73	8 MVA, 3 Fall	GCS: 8.4±4.0 (3–15)	Scan: both the acute (3.1±1.9 days) and subacute (27.6±23.4 days) stages; Scale: 124.7±96.0 days	DRS	CC	FA	Spearman
**ROI studies with WBA**												
	Bendlin et al., 2008	US	35/19	30.54±11.37/28.11±9.43	13.26±1.62 / 14.25±1.96	74.29	Rapid impact injury to the brain (such as a MVA or fall)	Moderate to severe	Visit1: average 56 days (28-81 days); Visit2: about 1 year(252-380 days)	GCS	CC, IC, SLF, ILF, CG, insula, hippocampus, CR, tr, whole-brain WM	FA, MD, WM volume	-
	Marquez et al., 2011	US	30/19	26.60±10.50 (16-57) / 28.68±10.13 (17-50)	11.83±2.56 / 15.72±3.14	70.00	Closed head traumatic brain injury through a mechanism consistent with TAI, such as high-speed MCV	Most were severe injuries (GCS 5.97±4.19); Moderate disability after 8 months: GOSE: 5.97±1.97	Scan: 7.88±1.86 months; Scale: 8 months after injury	GOSE	CC, whole-brain WM	FA, MD	Spearman
	Tang et al., 2012	US	12/11	39.6/40.1	-	-	-	Mild to severe	-	7-point scale	CC, whole-brain WM	FA, MD	-
**WBA studies**												
	Benson et al., 2007	US	20/14	35.5±14.6 (11-57) / 27.5±5.7 (23-45)	-	65.00	17 MCA, 2 Fall, 1 Assault	Mild to severe: GCS: 7.8±4.4 (3-15)	Scan: mean 35.3 months; Scale: at time of admission	GCS	Whole-brain WM	FA	Spearman

Newcombe et al., 2010	UK	12/32	43.14±19.54	-	71.43	3 MCA, 2 Fall, 2 Assault	Severe: VS	Scan and scale: 335.43±227.78 days (3-22 months)	CRS-R	Supratentorial central whole-brain WM	FA, ADC	Spearman

Exp, experimental group; Ctr, control group; MVA, motor vehicle accident; DAI, diffuse axonal injury; VS, vegetable state; TBI, traumatic brain injury; GCS, Glasgow Coma Scale; GCSE Glasgow Coma Scale-Extended; GOSE, Glasgow Outcome Scale-Extended; MVC, motor vehicle collision; TAI, Traumatic axonal injury; CC, corpus callosum; IC, internal capsule; WM, white matter; SLF, superior longitudinal fasciculus; ILF, inferior longitudinal fasciculus; PVF, peduncular projections to the ventral frontal cortex; PDF, peduncular projections to the dorsal frontal cortex; PPar, peduncular projections to the parietal cortex; POcc, peduncular projections to the occipital cortex; tr, thalamic radiation, CG, cingulate gurus, CR, corona radiate; WBA, whole brain analysis, FA, fractional anisotropy. The dash "-" represents "Not mentioned".

Supplementary Table S2. The DTI parameters of the included studies

Authors & Year	Manufacturers of scanners	Field strength (Tesla)	Sequence	TR/ TE ms	# Non-colinear directions	b values (sec/mm2)	# b0 images	Field of view (FOV) (mm×mm)	Matrix size (acquisition matrix)	In-plane resolution (mm×mm)	Section thickness (mm)	Gap (mm)	# Slices	Acquisition time (sec)
Huisman et al., 2004	-	1.5	T2-weighted, SE-EPI	6000/118	6	1221	3	400×200	256 × 128	-	6.0	-	-	126
Wang SK et al., 2005	Siemens	3.0	-	6000/103	16	1500		230×230		-	-	-	36	900
Benson et al., 2007	Siemens Sonata	1.5	T2-weighted, SE-EPI	5800/97	6	1000	0**	256×256	128 × 128	2 × 2	4.0	-	35	413
Bendlin et al., 2008	GE SIGNA MRI system	3.0	Cardiac-gated, diffusion-weighted, spin-echo, single-shot, EPI pulse	1000～1500*/78.2	12	1114		240×240	120 × 120	2 × 2	3.0	0	39	390～480*
Wang JY et al., 2008	GE Signa Excite	3.0	Single-shot, SE-EPI	12000/75.5	19	1000	3	240×240	128 × 128	2 × 2	3.0	0	45	540
Wang SK et al., 2008	Siemens Magnetom Trio	3.0	-	-	-	-	-	-	-	-	-	-	-	-
Wang BC et al., 2009	GE	1.5	Single-shot, SE-EPI	9000/83.2	25	1000	-	240×240	-	-	4.0	0		252
Newcombe et al., 2010	Siemens Magnetom Trio	3.0	-	8300/98	12	338～1588	5	192 × 192	96 × 96	-	2.0	-	63	-
Dong et al., 2011	GE Signa Exicite Ⅱ	1.5	Single-shot, EPI	9000/96	25	1000	-	-	-	-	5.0	0	28	-

Marquez et al., 2011	GE Signa Exicite	3.0	Single shot, SE-EPI sequence	12000/75.5	19	1000	3	240×240	128 × 128	2 × 2	3.0	0	45	540
Tang et al., 2012	Philips Achieva	3.0	Diffusion-weighted SE sequence	5682/70	32	1200	-	210×210	104 × 106	-	2.5	0	54	-
Zhang et al., 2012	GE Signa Exicite HD	1.5	Single-shot, SE-EPI	10000/95	25	1000	-	240×240	128 × 128	-	4.0	0	-	-
Arenth et al., 2014	Siemens Allegra	3.0	EPI sequences	6440/73	6	850	1	200×200	128 × 128	-	3.0	-	-	-
Haberg et al. , 2015	Siemens Trio	3.0	Single-shot balanced-echo EPI sequence	6800/84	30	1000	6	240×240	96 × 96	-	2.5	0	55	-
Yi et al., 2015	GE Signa Exicite HD	1.5	Single-shot, SE-EPI	-	15	1000	-	160×160	288 × 224	-	4.0	0	-	-
Edlow et al., 2016	GE Medical Systems	1.5	Single-shot/twice refocused, SE-EPI	5000-7500/72.9-99.4	6 or 25	1000	1 or 3	220×220	128 × 128	0.86 × 0.86 ～ 1.72 × 1.72	5.0	1 3	-	-

GE, General Electric; SE-EPI, spin-echo echo-planar imaging; SE, spin-echo; EPI, echo-planar imaging. *: dependent upon the heart rate; **: corresponding to T2-weighted images; #: number of.

Supplementary Table S3. Answers to signaling questions of QUADAS-2

	1 Patient Selection		2 Index Test		3 Reference Standard		4 Flow and Timing			
Study	Q1.1	Q1.2	Q2.1	Q2.2	Q3.1	Q3.2	Q4.1	Q4.2	Q4.3	Q4.4
Arenth et al. (2014)	N	Y	U	N	Y	Y	N	Y	Y	Y
Bendlin et al. (2008)	N	Y	U	N	Y	Y	N	Y	Y	N
Benson et al. (2007)	N	N	N	N	Y	Y	N	Y	Y	Y
Dong et al. (2011)	U	Y	U	N	Y	Y	Y	Y	Y	Y
Edlow et al. (2016)	U	N	Y	N	Y	U	N	N	Y	N
Haberg et al. (2015)	Y	N	U	N	Y	Y	Y	Y	Y	N
Huisman et al. (2004)	Y	Y	U	N	Y	Y	Y	Y	Y	Y
Marquez et al. (2011)	N	N	U	N	Y	Y	Y	Y	Y	Y

Newcombe et al. (2010)	N	Y	U	N	Y	Y	U	Y	Y	Y
Tang et al. (2012)	N	Y	U	N	Y	Y	U	Y	Y	Y
Wang BC et al. (2009)	U	Y	U	N	Y	Y	Y	Y	Y	Y
Wang JY et al. (2008)	U	Y	Y	N	Y	U	N	N	Y	N
Wang SK et al. (2005)	N	Y	U	N	Y	Y	Y	Y	Y	Y
Wang SK et al. (2008)	N	Y	U	N	Y	Y	Y	Y	Y	Y
Yi et al. (2015)	U	Y	U	N	Y	Y	Y	Y	Y	Y
Zhang et al. (2012)	Y	Y	U	N	Y	Y	Y	Y	Y	N

Y, yes; U, unclear; N, no; Q1.1-4.4 are listed below.

Note: Signaling questions in Table S2 (adapted for specific clinical theme):

Domain 1: Patient Selection

Q1.1: Was a consecutive or random sample of patients enrolled?

Q1.2: Did the study avoid inappropriate exclusions?

Domain 2: Index Test

Q2.1: Were the DTI scan results interpreted without knowledge of the results of the reference standard?

Q2.2: If a threshold was used, was it prespecified?

Domain 3: Reference Standard

Q3.1 Is the reference standard likely to correctly classify the degree of impaired consciousness?

Q3.2 Were the reference standard results interpreted without knowledge of the results of the DTI scan?

Domain 4: Flow and Timing

Q4.1 Was there an appropriate interval between index tests and reference standard?

Q4.2 Did all patients receive a reference standard?

Q4.3 Did all patients receive the same reference standard?

Q4.4 Were all patients included in the analysis?

(Answers to above questions can be yes, no, or unclear)
